# Myricetin protects against doxorubicin-induced acute kidney injury in rats by mitigating oxidative damage and apoptotic response

**DOI:** 10.3389/fphar.2025.1601628

**Published:** 2025-06-30

**Authors:** Muhammed Talha Karadogan, Betul Cicek, Kagan Tolga Cinisli, Ali Sefa Mendil, Mustafa Ozkaraca, Furkan Yilmaz, Halis Suleyman

**Affiliations:** ^1^ Department of Child Nephrology, Cerrahpasa Medical Faculty, Istanbul University, Istanbul, Türkiye; ^2^ Department of Physiology, Faculty of Medicine, Erzincan Binali Yildirim University, Erzincan, Türkiye; ^3^ Vaccine Development Application and Research Center, Ataturk University, Erzurum, Türkiye; ^4^ Department of Pathology, Veterinary Faculty, Erciyes University, Kayseri, Türkiye; ^5^ Department of Pathology, Veterinary Faculty, Cumhuriyet University, Sivas, Türkiye; ^6^ Department of Pharmacology, Faculty of Medicine, Erzincan Binali Yildirim University, Erzincan, Türkiye

**Keywords:** Acut kidney injury, antioxidant, apoptosis, doxorubicin, myricetin, oxidative stress, rat

## Abstract

**Introduction:**

Doxorubicin (DOX) is a potent anti-neoplastic agent widely preferred in treating various tumors. However, DOX’s off-target toxicity in healthy tissues, such as nephrotoxicity, limits its clinical utilization. DOX generates oxidative stress and apoptosis in the kidneys, which stimulates cytotoxic cellular signaling. Myricetin (MYC), an important natural flavonoid, exhibits antioxidant and antiapoptotic features. In this regard, the current report was designed to explore the renoprotective potential of MYC on DOX-induced nephrotoxicity.

**Methods:**

Animals were divided into four groups with six rats in each group: control, MYC, DOX, MYC + DOX. MYC was given orally to rats at 100 mg/kg for 10 days and DOX was injected intraperitoneally as a single dose of 20 mg/kg on the eighth day. Serum samples were evaluated for creatinine and blood urea nitrogen (BUN), and histopathological analysis of the kidneys was conducted. The levels of malondialdehyde (MDA), myeloperoxidase (MPO), total oxidant status (TOS), glutathione (GSH), glutathione peroxidase (GPx), and total antioxidant capacity (TAC) were measured in the renal tissues. Additionally, biochemical assessments of Bax and Bcl-2 proteins, along with immunohistochemical evaluations of the expression levels of caspase-3 and apoptosis-inducing factor (AIF), were conducted to evaluate apoptosis.

**Results:**

Pre-treatment of MYC decreased DOX-elicited elevation in creatinine and BUN levels (p < 0.05). Histopathological findings demonstrated the nephroprotective role of MYC on renal damage (p < 0.05), which was in harmony with the biochemical findings. Furthermore, MYC demonstrated antioxidant properties by reversing the increase in MDA, TOS, and MPO levels and the decrease in GSH, GPx, and TAS levels caused by DOX (p < 0.05). MYC pre-treatment also markedly prohibited DOX-induced elevation of Bax level, and rise of expression of caspase-3 and AIF, and reduction of Bcl-2 levels (p < 0.05).

**Conclusion:**

It could be supposed that the nephroprotective role of MYC towards DOX-induced kidney damage might be mediated by its antioxidant and antiapoptotic features.

## Introduction

Doxorubicin (DOX) is an anthracycline antibiotic, frequently used to treat many cancer types (Sun et al., 2024). However, the toxicity of DOX on non-target organs and tissues severely limits its clinical application (Kciuk et al., 2023). DOX tends to accumulate in the glomerulus, which is associated with glomerular and tubular toxicity (Lahoti et al., 2012; Suleimani et al., 2023). It is suggested that patients develop nephrotic syndrome with renal lesions and focal segmental glomerular sclerosis following treatment with DOX (Santos et al., 2020). DOX-iron complexes generate ROS release and lipid peroxidation (LPO), depletion of cellular antioxidants, and protein/nucleic acid damage (Cirrik et al., 2023; Owumi et al., 2021). Remarkably, it has been emphasized that DOX-associated oxidative stress activates mitochondrial permeability and apoptogenic signaling pathways (Vitale et al., 2024). The increase of proapoptotic proteins (Bcl-2–associated X protein [Bax], apoptosis-inducing factor [AIF], and caspase-3) and reduction of antiapoptotic protein B-cell lymphoma-2 [Bcl-2] activation can also be induced by DOX, which leads to tissue apoptosis (Arunachalam et al., 2022; Sangweni et al., 2022). Despite extensive research, protective measures and new treatment strategies against the DOX-related nephrotoxicity remain limited. The use of antioxidant compounds derived from plants is supported by various research studies because of their non-toxic nature, their capability to reduce the toxic effects of chemotherapy, and their relatively low cost (Abe et al., 2018).

Myricetin (MYC) is a natural flavonoglycoside derived from the fruits, leaves, branches and peels of other natural plants (Sun et al., 2016). This phenolic compound is commonly found in various fruits and vegetables, as well as some types of teas produced from plants. It exists in both free and glycosidic bound forms (Semwal et al., 2016). Myricetin is structurally related to several phenolic compounds, namely, quercetin, morin, and kaempferol (Imran et al., 2021). Additionally, the compound is also referred to as hydroxyquercetin, because of its structural resemblance to quercetin (Umadevi et al., 1988). Evidence has reported that MYC displays a wide range of pharmacological activities, including antioxidant (Imran et al., 2021), anti-inflammatory (Liao et al., 2019), and anti-apoptotic (Guo et al., 2024). Recently, MYC has been shown to induce the production of antioxidant enzymes that cleanse ROS and protect cells against oxidative stress (Wang et al., 2010). Moreover, it has been documented that MYC has an antiapoptotic effect by inhibiting the mitochondria-induced apoptosis pathway caused by H_2_O_2_ in cells (Kang et al., 2010). With all these properties, it can serve as a specific protective agent against kidney damage (Alaryani, 2024). Although MYC has been demonstrated to have promising protective and therapeutic potential in various experimental models of renal injury (Jee et al., 2020; Yuan et al., 2024), its effect on DOX-induced nephrotoxicity has not been investigated. This gap in the literature is the impetus for our investigation into the protective potential of MYC against DOX-induced renal injury.

Based on the foregoing information, the present research aimed to investigate whether MYC can protect against acute nephrotoxicity induced by DOX in the rat model. It was hypothesized that MYC would protect from DOX-evoked renal oxidative injury and proapoptotic response, eventually improving renal function and reducing kidney damage. To test our hypothesis, we evaluated renal function by measuring creatinine and blood urea nitrogen (BUN) levels, as well as histopathological analyses of kidney tissues. It was also determined malondialdehyde (MDA), myeloperoxidase (MPO), total oxidant status (TOS), glutathione (GSH), glutathione peroxidase (GPx), and total antioxidant status (TAS) to assess the protective efficacy in oxidative response. Additionally, biochemical assessments of Bax and Bcl-2 proteins, along with immunohistochemical evaluations of the expression levels of caspase-3 and AIF, were conducted to evaluate apoptosis to better understand the underlying mechanisms of the protective’s impacts.

By performing this research, we hope to contribute to identifying MYC as a promising protective candidate against the nephrotoxicity led by DOX through its antioxidant and antiapoptotic features.

## Materials and methods

### Chemicals

The chemotherapeutic drug doxorubicin hydrochloride (50 mg/25 mL) used in this study was obtained from Saba drug industries (Adrimisin^®^, İstanbul, Turkey)', MYC (Catalog no. 476275, 96% purity) Sigma-Aldrich (Darmstadt, Hesse, Germany). Thiopental sodium (0.5 g vial), was acquired from, I.E., Ulagay (Istanbul, Turkey).

## Animals

In this study, 24 albino Wistar male rats weighing 260 ± 30 g (10–12 weeks) were used. Experimental animals were obtained from Erzincan Binali Yıldırım University Experimental Animals Application and Research Center (EBYÜ-DEHAM). All rats were randomly divided into experimental groups and housed in groups in conventional cages. The animals were fed *ad libitum* with standard laboratory chow and tap water. The laboratory environment in which the rats were housed was set at 22 ±1°C temperature and 50%–65% relative humidity. The laboratory rooms were automatically set to a 12-h light-dark cycle suitable for the physiology of the rats. The 3R principles were adhered to during the planning and conduct of the experiment. Experimental applications were executed following the International Guide for the Use and Care of Laboratory Animals (ARRIVE).

### Experimental induction and drug pre-treatment of DOX-Induced renal toxicity in rats

DOX-evoked renal toxicity induction was performed by Chen et al. using the method described earlier (Chen et al., 2016). In our study, we preferred the acute toxicity (short-term model) model created with a single dose of DOX. In this model, it has been reported that a single DOX dose (usually ranging from approximately 5–30 mg/kg DOX dose) can subsequently cause oxidative liver, kidney, and heart damage (AlAsmari et al., 2021; Chen et al., 2016; Podyacheva et al., 2021). In addition, the single dose of 20 mg/kg we employed in the current report corresponds to a high single dose used to treat cancer patients in the clinic (Šimunková et al., 2020). MYC dose was selected according to previous studies in the literature (Berköz et al., 2024). Doxorubicin hydrochloride was dissolved in 0.9% normal saline to achieve a concentration suitable for intraperitoneal administration at a dose of 20 mg/kg. Myricetin was initially dissolved in a minimal volume of dimethyl sulfoxide (DMSO; final concentration <5% v/v) and then diluted with 0.9% normal saline to reach the required concentration for 100 mg/kg dosing. For both compounds, the injection volumes were adjusted such that each animal received 1.0 mL of the respective solution, corresponding to the target dose per kilogram of body weight. Control and DOX-only groups received 1.0 mL of the appropriate vehicle solution to ensure volume consistency across all experimental groups.

## Experimental design

**FIGURE 1:**
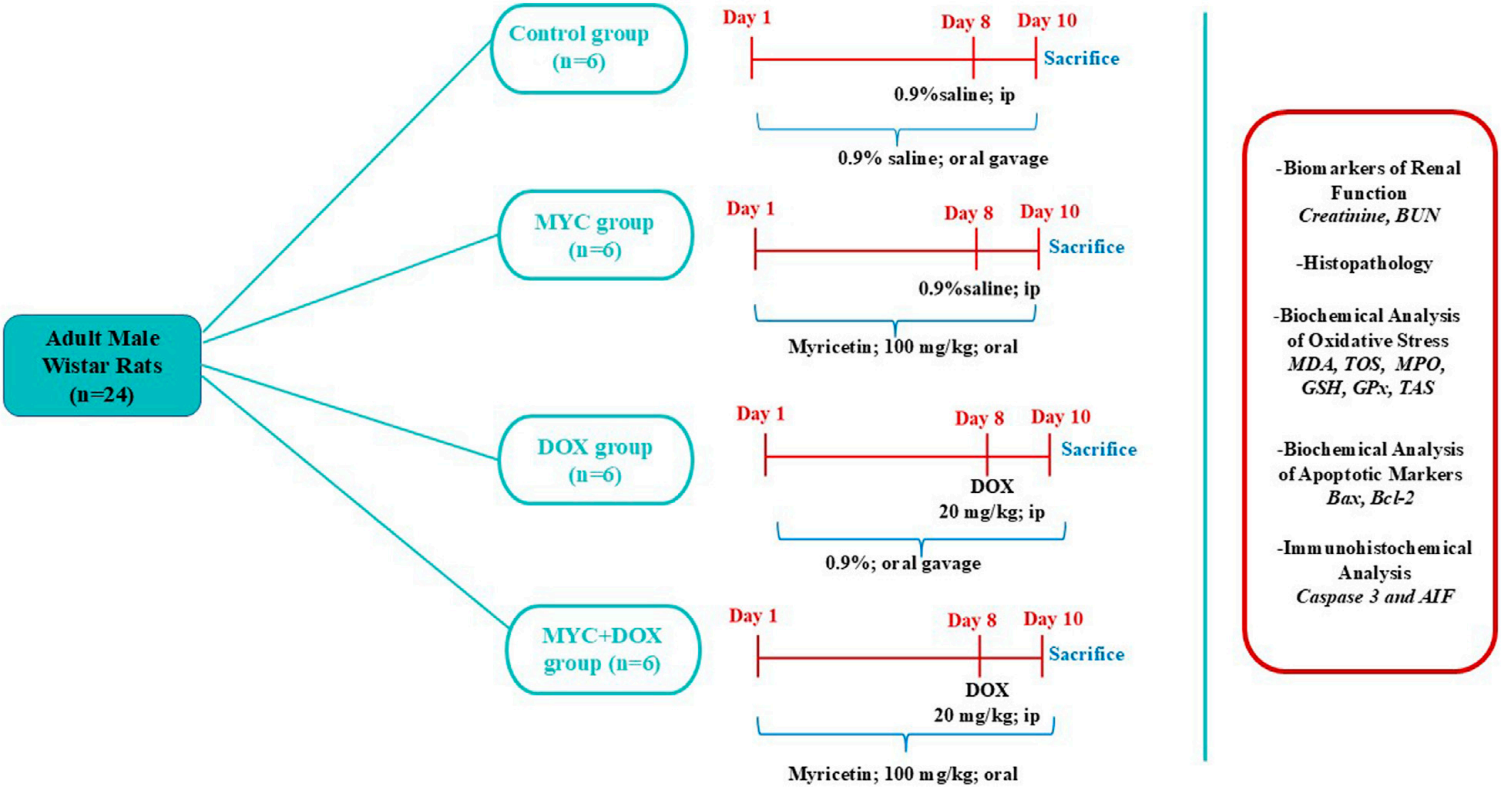
Experimental Schedule. DOX,doxorubicin; MYC, myricetin, BUN, Blood urea nitrogen; MDA, malondialdehyde; TOS, total oxidant status; GSH, glutathione; GPx, glutathione peroxidase, TAS, total antioxidant status; MPO, myeloperoxidase; AIF, apoptosis-inducing factor; Bax; Bcl-2–associated X protein, Bcl-2; antiapoptotic protein B-cell lymphoma-2.

The rats were divided into four groups at random, each consisting of six rats. (I) Control; 0.9% normal saline was given via oral gavage for 10 days and 0.9% normal saline was injected intraperitoneally (i.p) on day 8, (II) MYC group was treated with 100 mg/kg of MYC with oral gavage for 10 days without DOX administration, (III) DOX group; 0.9% normal saline was given via oral gavage for 10 days and was administered a single i. p injection of 20 mg/kg DOX on eighth day, (IV) MYC + DOX group were treated with 100 mg/kg MYC for 10 days and a single dose of 20 mg/kg DOX was given as i. p on eighth day. The experiment’s schema was shown in Figure 1.

### Collection of blood samples and renal tissues

On the 10th day of experiment, 30 min after administration of last dose of MYC, all rats were anesthetized with sodium thiopental (50 mg/kg, i. p) (Chen et al., 2016; Okkay et al., 2022). The whole blood sample was taken directly from the heart with a 21 G injectable needle and 5 mL syringe without damaging the heart tissue. Immediately after cervical dislocation, a long surgical incision was made on the ventral surface of the thorax and abdomen, the abdomen organs were exposed, and kidney tissues were dissected. These tissues were washed with phosphate-buffered saline (PBS) after dissection. 1M PBS (pH = 7.4) was added at the rate of 1 g of tissue/9 mL PBS and kidney tissues were homogenized on ice. After that, it was centrifuged at a speed of 5,000 rpm for 20 min at a temperature of +4 C. The supernatants were then collected and stored at −80°C for various biochemical tests. The collected blood samples were rested at room temperature for 20 min, then centrifuged at 15,000 rpm at 4°C for 15 min. Renal function tests were performed in the collected serum samples.

### Measurement of kidney function parameters

Serum BUN and creatinine levels were measured spectrophotometrically using a Roche cobas^®^ 8,000 autoanalyzer (Mannheim, Germany). BUN was calculated using the following formula: BUN = URE x 0.48). This kinetic colorimetric assay was based on the Jaffe method (Moore and Sharer, 2017). Creatinine forms a yellow-orange complex with picrate in an alkaline solution. The intensity of the color formed is measured photometrically (at 505 nm), and the creatinine concentration is directly proportional to the color intensity. Analyses using the rate-emptying method minimize inhibition by bilirubin. Serum samples contain proteins that react nonspecifically according to the Jaffe method. Urea is hydrolyzed by urease to form CO^2^ and ammonia. The resulting ammonia then reacts with α-ketoglutarate and reduced nicotinamide adenine dinucleotide (NADH) in the presence of glutamate dehydrogenase to produce glutamate and nicotinamide adenine dinucleotide (NAD^+^). The decrease in absorbance caused by the reduction of the NADH complex is determined by a photometric method (at 340 nm) (Dagel et al., 2024).

### Histopathological method

Kidney tissues obtained from rats by necropsy were fixed in a 10% neutral formalin solution. The tissues were then subjected to a 18-h wash in tap water. For the purpose of dehydration, the tissues were immersed in 70%, 80%, and 90% ethanol for a period of 2 hours for each respective concentration. The samples were then dehydrated by means of immersion in absolute ethanol. The tissues were then subjected to a process of clearing, which involved a series of washes in xylene (1.5 h, twice). The tissues were embedded in paraffin blocks by incubating them in xylene-paraffin at 60°C for 1.5 h and then in pure paraffin at 60°C for a further 1.5 h. Six distinct sections (4 µm) were obtained from the kidney tissues of each rat. The sections were stained with Mayer’s hematoxylin solution (Cat. No. MHS1, Sigma Merck, Darmstadt, Germany). Counterstaining was then carried out with eosin solution (Cat. No. 318906, Sigma Merck, Darmstadt, Germany). The stained sections were examined under a light microscope (Cat. No. DM2500, Leica, Wetzlar, Germany). The evaluation was performed semi-quantitatively in six randomly selected areas according to a modified version of the method described by Chen et al. (2016). All rats were included in the histopathological evaluation. Six kidney sections were taken from each rat and six images were obtained from each section. Sections were graded for necrotic-degenerative changes and interstitial nephritis as follows: Absent, mild (5%–10%), moderate (11%–20%), and severe (>21%) were the categories employed (Chen et al., 2016).

### Evaluation of renal oxidative stress and apoptotic parameters

The MDA level was evaluated using the method developed by Ohkawa et al. (Ohkawa et al., 1979). This method is based on the measurement of the pink complex formed aftermath of the MDA and thiobarbituric acid reaction in a spectrophotometer at 532 nm.

GSH concentrations were determined by the modified Ellman method and the yellowish-green complex formed as a result of the reaction was spectrophotometrically measured at 412 nm (Beutler, 1975).

GPx activity was evaluated according to the Paglia and Valentine method, in which the absorbance converted throughout oxidation of NADPH to NADP+ was measured in the range of 340 nm (Paglia and Valentine, 1967).

TAS (Catalog no. RL0017) was determined using a new automated measurement method and kit (Rel Assay Diagnostics, Mega Tıp, Gaziantep, Turkey). The test is related to antioxidants in the spe The alteration of absorbance at 660 nm is correlated with total antioxidant concentration of the samplecimen that diminishes dark blue-green ABTS radical to an uncoloured reduced ABTS form. (Erel, 2004).

The measurement of serum TOS (Catalog no. RL0024) concentration was determined via an automatic method developed by Erel (Rel Assay Diagnostics, Mega Tıp, Gaziantep, Turkey) (Erel, 2005). The test is associated with oxidants available in the specimen oxidize the ferrous ion-chelator complex to ferric ion. The ferric ion forms a colored complex with chromogen in an acidic environment. The color intensity, which can be determined with a spectrophotometer, is linked to the total amount of oxidant molecules existent in the specimen.

The absorbance of the yellowish-orange complex that results from MPO oxidizing o-dianicide in the presence of hydrogen peroxide at a wavelength of 460 nm was measured kinetically to determine the MPO level (Bradley et al., 1982). The method of Lowry et al. was used to determine the level of protein content in tissues (Lowry et al., 1951).

Bax (Catalog no. 201-11–0035) and bcl-2 (Catalog no. 201-11–0038) levels in kidney tissues were performed using commercial ELISA test kits (Sunred Biological Technology, Shanghai, China). The manufacturer’s instructions were followed when conducting the test analyses. Briefly, standards and samples were added standard and sample wells, respectively. Streptavidin-HRP was put into each well and incubated for 1 h at 37°C. Then, the plate was washed and added substrate solutions A and B for 10 min to incubate, and stop solution was added. Bax and Bcl-2 concentrations in the samples were evaluated from their interrelated absorbance values through the standard curve. Findings were normalized to total tissue protein and given as ng/mg protein.

### Estimation of immunohistochemical expression of cleaved caspase-3 and AIF

Kidney tissues were immunohistochemically stained with cleaved caspase three and AIF. For this purpose, 4 μm sections taken on polycicine slides were passed through xylol and alcohol series, and after washing with PBS, 3% H_2_O_2_ (Cat. no. 925B, Sigma Merck, Darmstadt, Germany) was kept for 10 min to ensure endogenous peroxidase inactivation. It was treated at 2 × 5 min 500 W with antigen retrieval solution to expose the antigen in the tissues. Then cleaved caspase 3 (Cat. no. E-AB-30004, Elabscience, Houston, Texas, United States) and AIF (Cat. no. Bs-0037R, Bioss, Woburn, Massachusetts, United States) was incubated overnight with primary antibodies (dilution 1/200). Secondaryly; Large Volume Detection System: anti-Polyvalent, HRP (Cat. no: TP-125-HL, Thermofischer, Waltham, Massachusetts, United States) was applied as recommended by the manufacturer. DAB 3-Amino-9-Ethylcarbazole (AEC) (Cat. no. TA-125-SA, Thermofischer, Waltham, Massachusetts, United States) was used as chromogen. After contrasting paint was made with Mayer’s Hematoksilen (Cat. no. MHS1, Sigma Merck, Darmstadt, Germany), it was covered with a water-based adhesive and inspected with a light microscope (Cat No. DM2500, Leica, Wetzlar, Germany). No immunoreactivity (0), mild (1), moderate (2), and severe (3) were assessed during the examination. According to the modification of the method described by Kucukler et al., immunreactivity was evaluated as non-semiquantitative (0), mild (+, 5%–10%), moderate (++, 11%–20%), severe (+++, 21%>) based on observations from six randomly selected microscopic fields (Kucukler et al., 2021).

### Statistical analysis

Statistical assessments were performed in the IBM SPSS Statistical Program for Windows version 22.0. (IBM Corp., Armonk, NY, United States). Data are presented as mean ± standard deviation (X ± SD). The conformity of quantitative data to normal distribution was confirmed by Shapiro Wilk test. A one-way ANOVA was used for analysis. According to the Levene test results, the Tukey or Games Howell tests was preferred as a *post hoc* test. Semi-quantitative histopathological and immunohistochemical data were analyzed by Kruskal Wallis test and *post hoc* Mann-Whitney U test. P less than 0.05 was regarded as statistical importance.

## Results

### Blood serum BUN and creatinine analysis results

**FIGURE 2:**
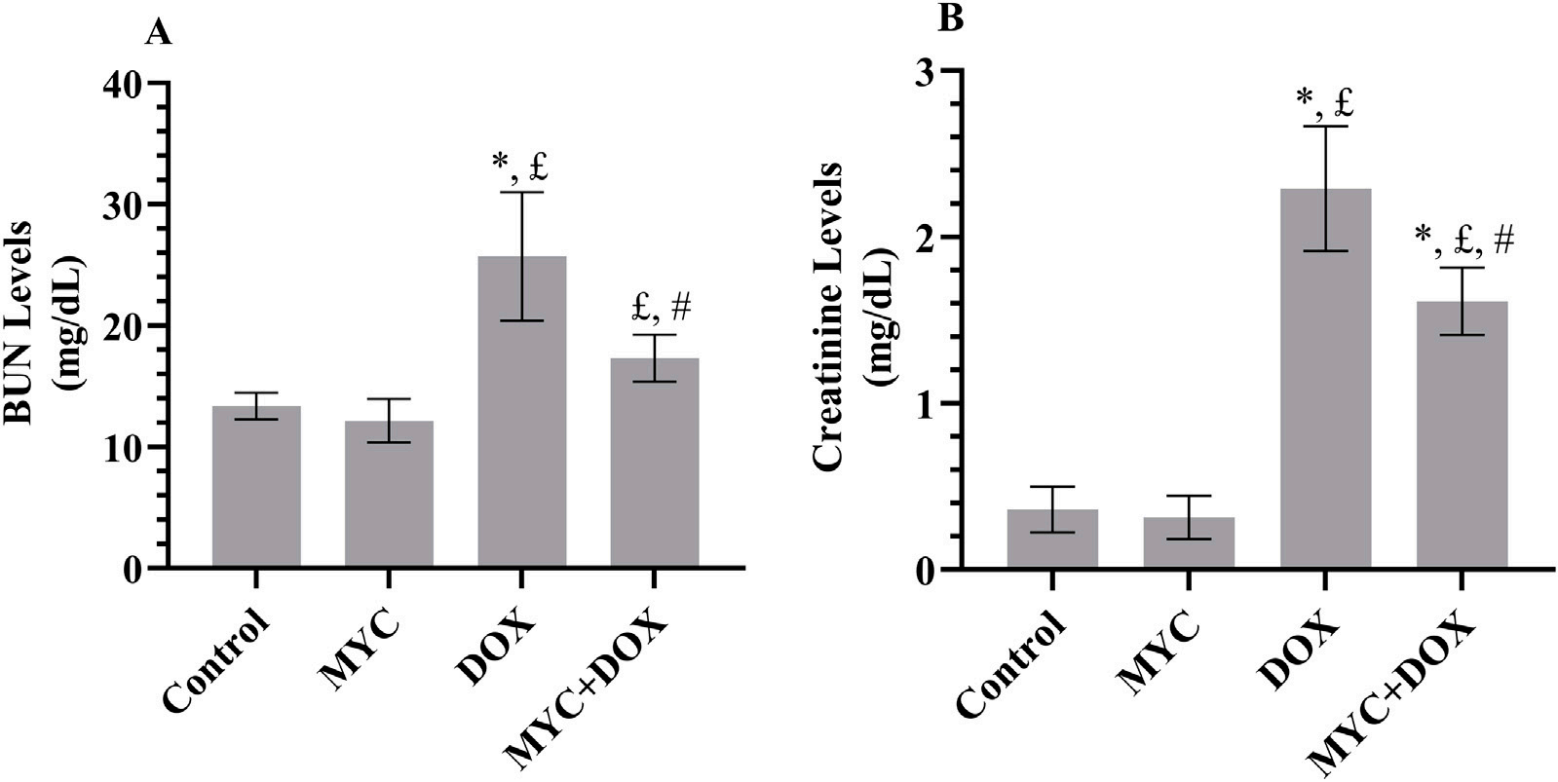
**(A, B)** Effect of DOX and MYC pre-treatment on BUN **(A)** and creatinine **(B)** levels on DOX administration. Results portrayed mean ± SD of six animals per group. *; p < 0,001 vs. control, £; p < 0.05 vs. MYC group, #; p < 0.05 vs. DOX group. BUN, Blood urea nitrogen; DOX, doxorubicin (20 mg/kg); MYC, myricetin (100 mg/kg).

In the current research, we evaluated the serum levels of BUN and creatinine as indicators of renal function. The results demonstrated a significant increase in serum BUN levels of 96% (p < 0.001, Figure 2A) and creatinine levels of 536% (p < 0.001, Figure 2B) in the DOX group compared to the control group. In the MYC + DOX group, a 36% (p = 0.001) and 45% (p = 0.003) decrease in BUN and creatinine levels, respectively, was observed when compared to the DOX group. Despite the BUN values being 29% higher in the MYC + DOX group compared to the control group, no statistical difference was identified (p = 0.139). Despite the observation that BUN levels were 9% lower and creatinine levels were 14% lower in the MYC group compared to the control group, no statistical difference was identified (p > 0.05).

### Histopathologic results

**FIGURE 3:**
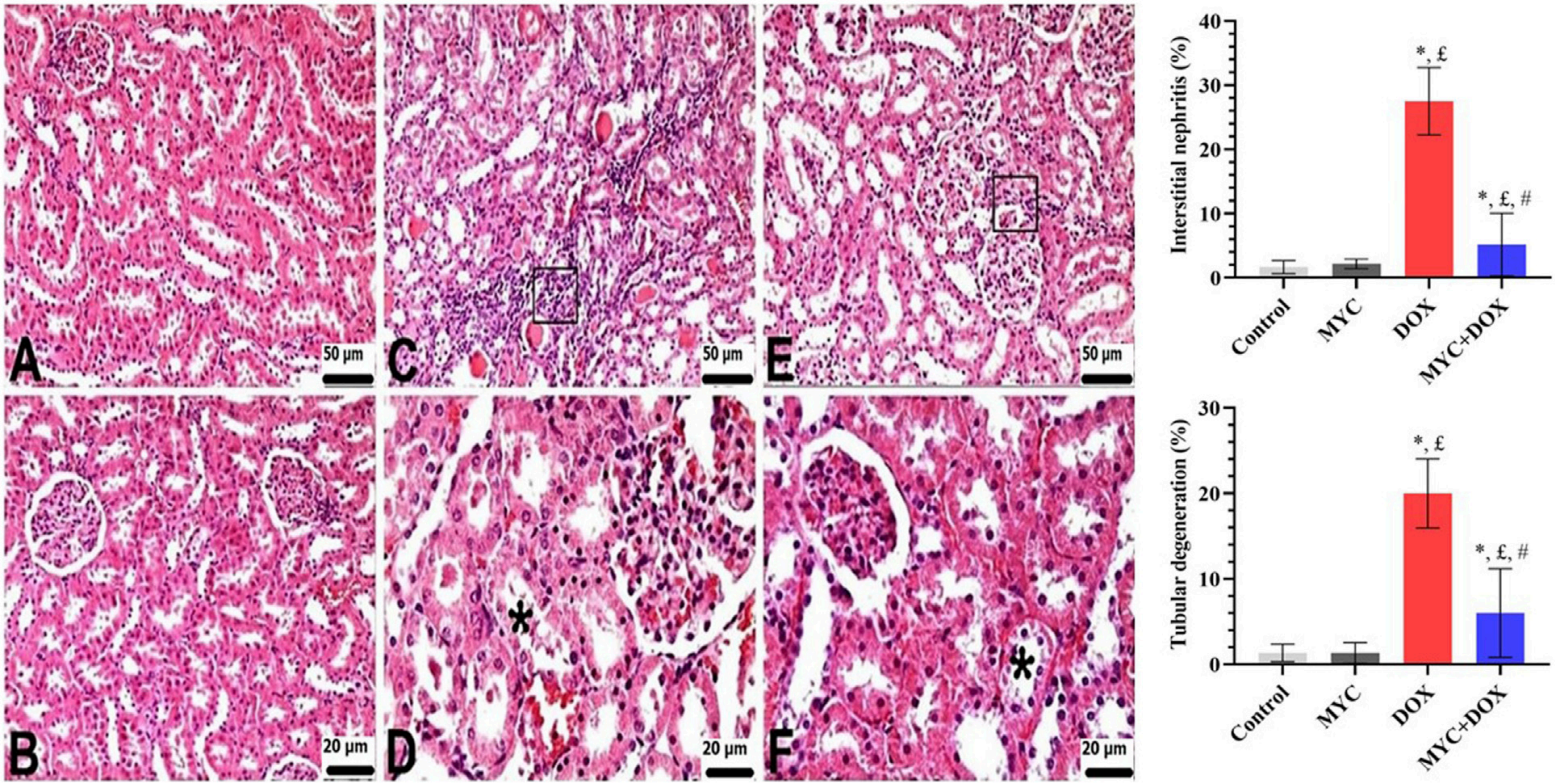
**(A–F)** Renal histological alterations of rat kidneys were observed using H&E staining. **(A,B)** Control **(A)** and MYC-only **(B)**. Normal kidney histology was observed **(C,D)**. DOX group. Disorganization of the normal renal architecture distorted by DOX administration was detected. Severe mononuclear cell infiltrations in interstitial areas (square) and moderate tubular degeneration (star). **(E,F)** MYC + DOX group. Pre-treatment with MYC reverted with these alterations, with few mononuclear cell infiltrations (square) and mild tubular degenerations (star) in interstitial areas. *; p < 0,05 vs. control, £; p < 0.05 vs MYC group, #; p < 0.05 vs. DOX group. Results portrayed mean ± SD of six animals per group. DOX,doxorubicin; MYC, myricetin.

According to the histopathological analysis, the renal tissues of rats in the control (Figure 3A) and MYC-only treated (Figure 3B) groups exhibited typical renal morphology and histological architecture. However, as visible in Figures 3C,D; DOX group displayed interstitial nephritis which was characterized by severe mononuclear cell infiltration, and tubular degeneration. In the kidney sections of the DOX group, interstitial nephritis was observed in an area corresponding to 27.5%, and tubular degeneration was observed in 20% of the area. In contrast, sections from the DOX group treated with MYC revealed a worthy of attention improvement in the tissue, with mild mononuclear cell infiltration and mild tubular degeneration (Figures 3E,F). A significant difference was detected between the MYC + DOX group and the DOX group in terms of interstitial nephritis and tubular degeneration (p < 0.05). Interstitial nephritis was found to have decreased by 82% in the MYC + DOX group in comparison with the DOX group, falling to 5%; tubular degeneration was found to have decreased by 70%, falling to 6%.

### MYC pre-treatment abates DOX-elicit renal oxidative damage in rats

**FIGURE 4:**
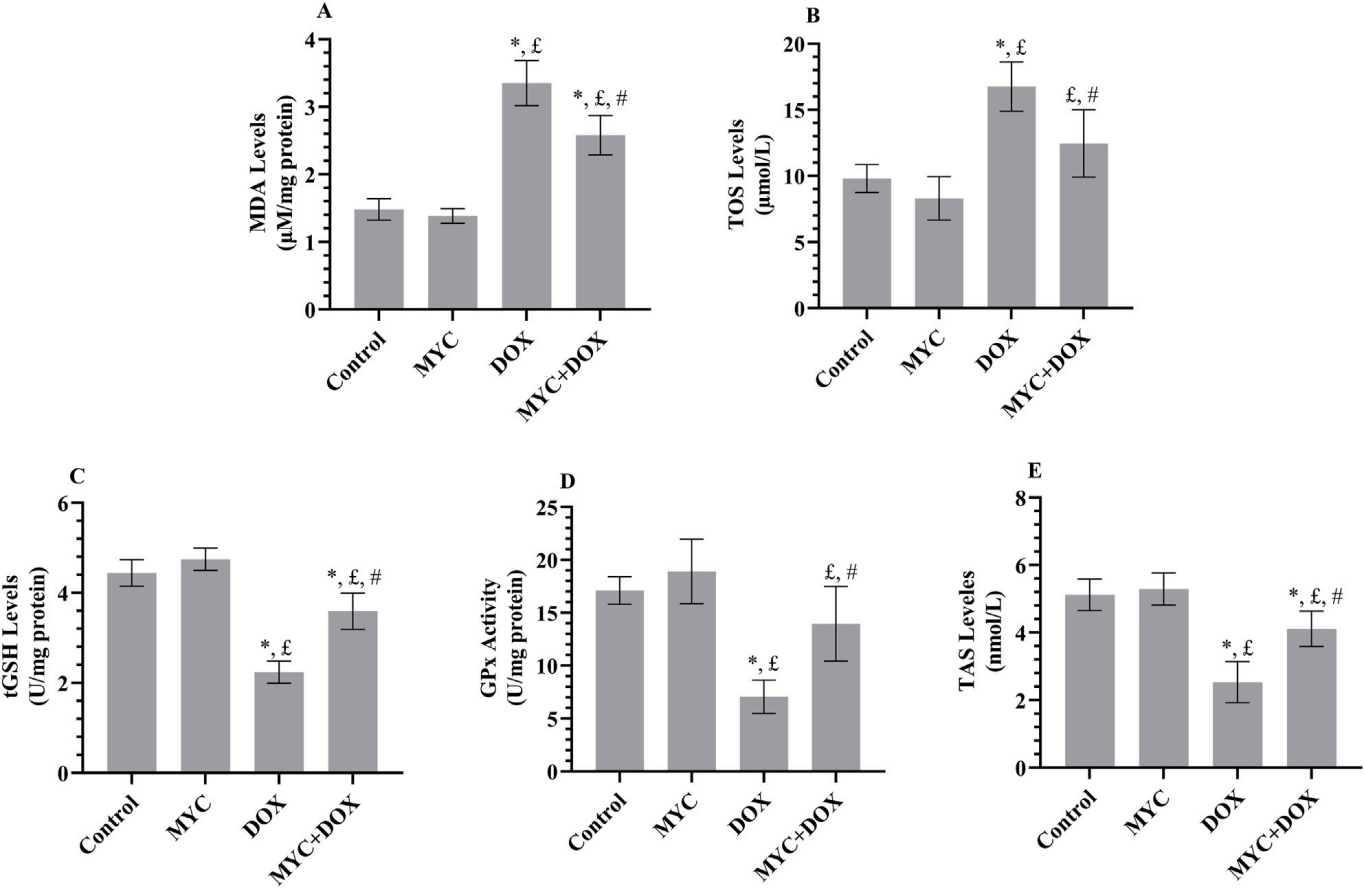
**(A–E)** Results of pre-treatment of MYC on MDA **(A)**, TOS **(B)**, GSH **(C)**, GPx **(D)**, and TAS **(E)** levels on DOX administration in the kidney tissue of rats. Results portrayed mean ± SD of six animals per group. *; p < 0,05 vs. control, £; p < 0.05 vs. MYC group, #; p < 0.05 vs. DOX group. MDA, malondialdehyde; TOS, total oxidant status; GSH, glutathione; GPx, glutathione peroxidase, TAS, total antioxidant status; DOX, doxorubicin (20 mg/kg); MYC, myricetin (100 mg/kg).

The protective sequel of MYC against DOX-related oxidative toxicity in rats’ kidneys is presented in Figures 4A–E. The DOX group demonstrated a 137% increase in MDA formation (p < 0.001) and a 71% increase in TOS level (p < 0.001) in comparison with the control group. Levels of the MDA in renal tissues from the MYC + DOX group were 33% lower than in the control group (p = 0.002; Figure 4A). TOS levels were 26% lower in the MYC + DOX group than in the control group (p = 0.004; Figure 4B). Despite the fact that the amount of TOS in the MYC + DOX group was 27% higher than in the control group, this difference was ultimately deemed to be of no statistical significance (p = 0.095). In terms of MDA and TOS, MYC group data were 7% and 15% lower than control group, respectively, but there was no statistical difference (p > 0.05).

When compared to the control group, the DOX group showed a 49% decrease in GSH levels (p < 0.001; Figure 4C) and a 59% decrease in GPx activity (p < 0.001; Figure 4D). In the MYC + DOX group, GSH levels were 60% higher (p < 0.001) and GPx activity was 98% higher (p = 0.001) compared to the DOX group. GPx activity in the MYC + DOX group was only 18% lower than in the control group (p = 0.176). In the MYC group, GSH data demonstrated an 8% increase, while GPx data exhibited an 11% increase in comparison with the control group (p > 0.05).

Besides, it was found that the kidney TAS level in the DOX group was 51% lower than that in the control group (p < 0.001; Figure 4E). However, it was observed that TAS levels increased by 63% in rats in the MYC + DOX group that received MYC prior to DOX treatment (p < 0.001). TAS levels were similar in the control and MYC groups, with only a 3% increase in MYC data (p = 0.936).

### MYC reduces the DOX-mediated elevation in MPO in kidney tissues of rats

**FIGURE 5:**
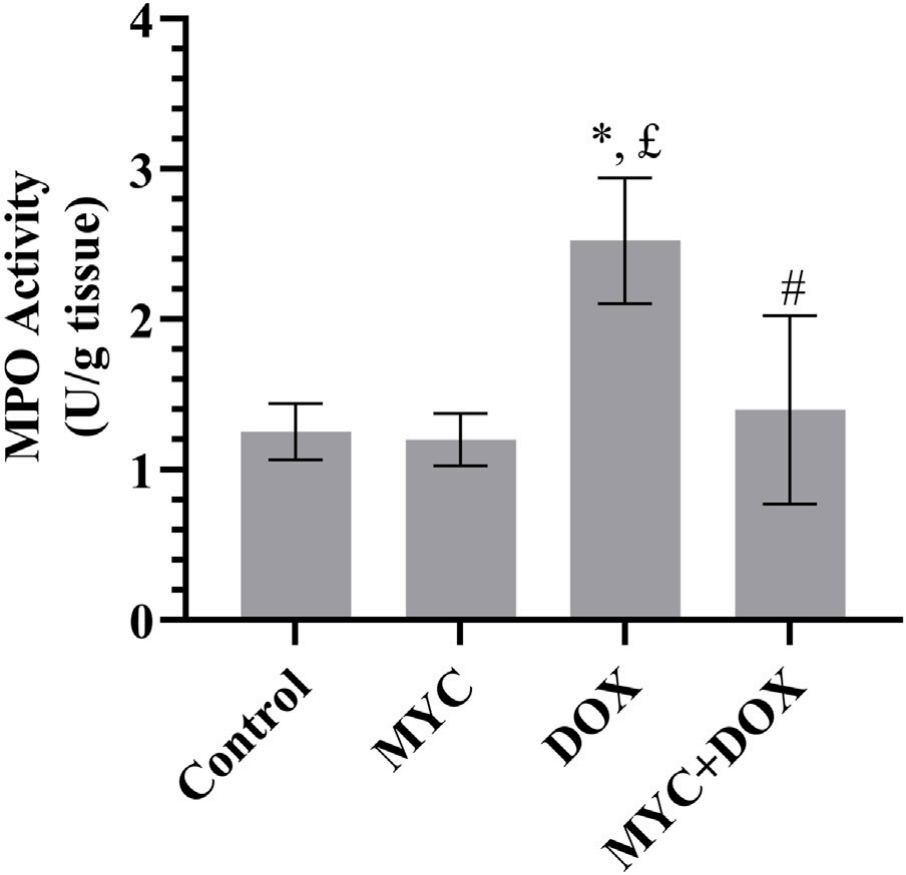
Effect of MYC on MPO level DOX administration in the kidney of rats. Results portrayed mean ± SD of six animals per group. *; p < 0,05 vs control, £; p < 0.05 vs. MYC group, #; p < 0.05 vs. DOX group. MPO, myeloperoxidase; DOX, doxorubicin (20 mg/kg); MYC, myricetin. (100 mg/kg).

The effects of MYC against DOX-mediated elevation in inflammation was presented in Figure 5. Herein, we examined MPO levels in renal tissue. MPO concentration increased by 102% in the DOX group compared to the control group (p = 0.001). MPO activity in rats in the MYC + DOX group was found to be 44% lower than in the DOX group (p = 0.024). MPO activity in the MYC + DOX group was only 12% higher than in the control group (p = 0.943). MPO activity in the MYC group was determined to be 5% lower than in the control group (p = 0.954).

### MYC reverse DOX-related apoptotic changes in renal tissue

**FIGURE 6:**
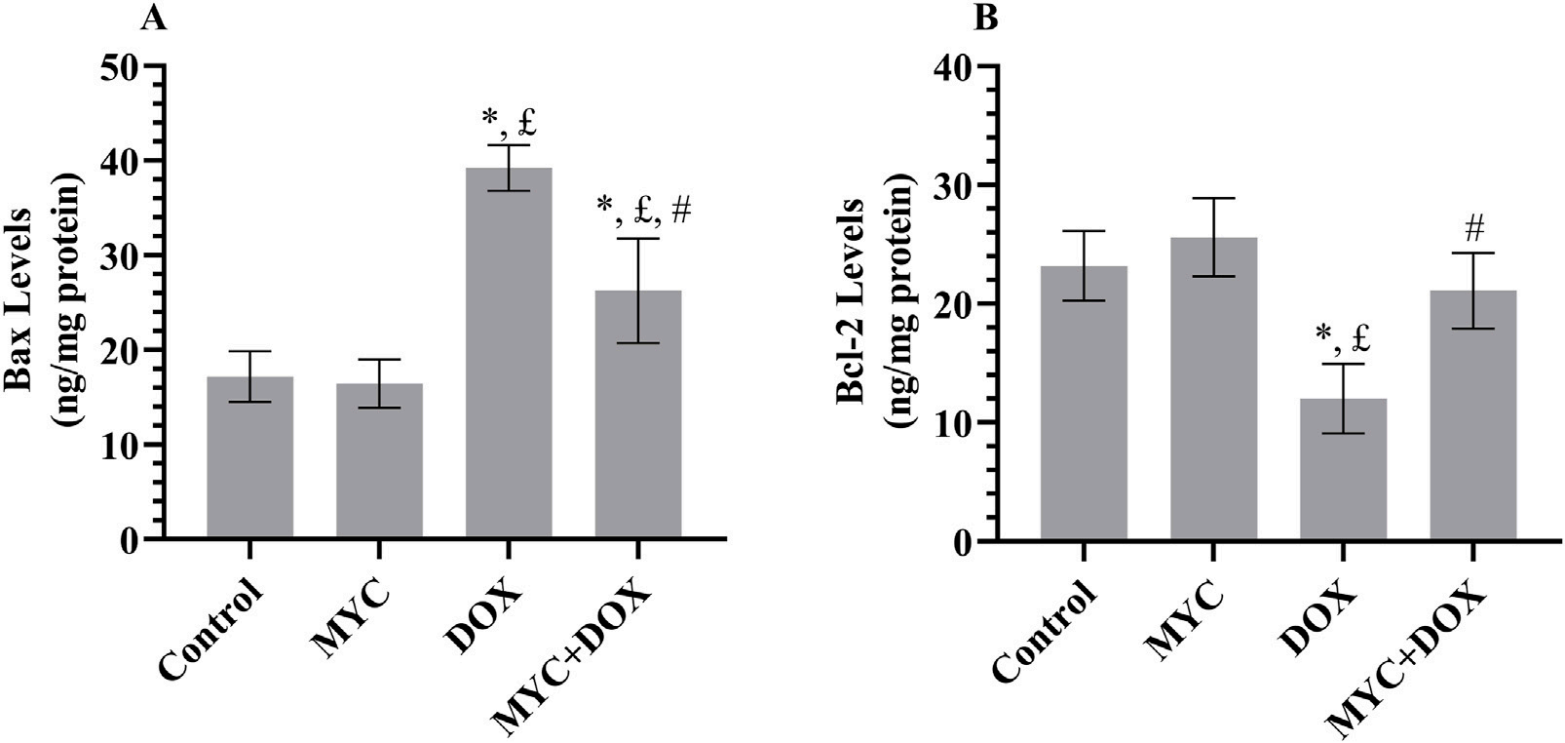
**(A,B)** MYC inhibits DOX-elicited apoptotic injury. Biochemical findings of Bax **(A)** and Bcl-2 **(B)** in renal tissue of rats from control, MYC, DOX, and MYC + DOX groups. Results portrayed mean ± SD of six animals per group. *; p < 0,05 vs. control, £; p < 0.05 vs. MYC group, #; p < 0.05 vs. DOX group. Bax; Bcl-2–associated X protein, Bcl-2; antiapoptotic protein B-cell lymphoma-2, DOX, doxorubicin (20 mg/kg); MYC, myricetin (100 mg/kg).

In the DOX group, a 128% increase in proapoptotic protein Bax levels (p < 0.001; Figure 6A) and a 48% decrease in antiapoptotic protein Bcl-2 levels (p < 0.001; Figure 6B) were determined compared to the control group. Remarkably, in the MYC + DOX group, Bax levels were 32% lower and Bcl-2 levels were 76% higher than in the DOX group (p > 0.001). Bcl-2 levels in the MYC + DOX group were only 9% lower than in the control group (p = 0.655). In the MYC group, Bax levels were 4% lower and Bcl-2 levels were 9% higher than in the control group, but there was no statistical difference between the two groups (p > 0.05).

### Immunohistochemical results

**FIGURE 7:**
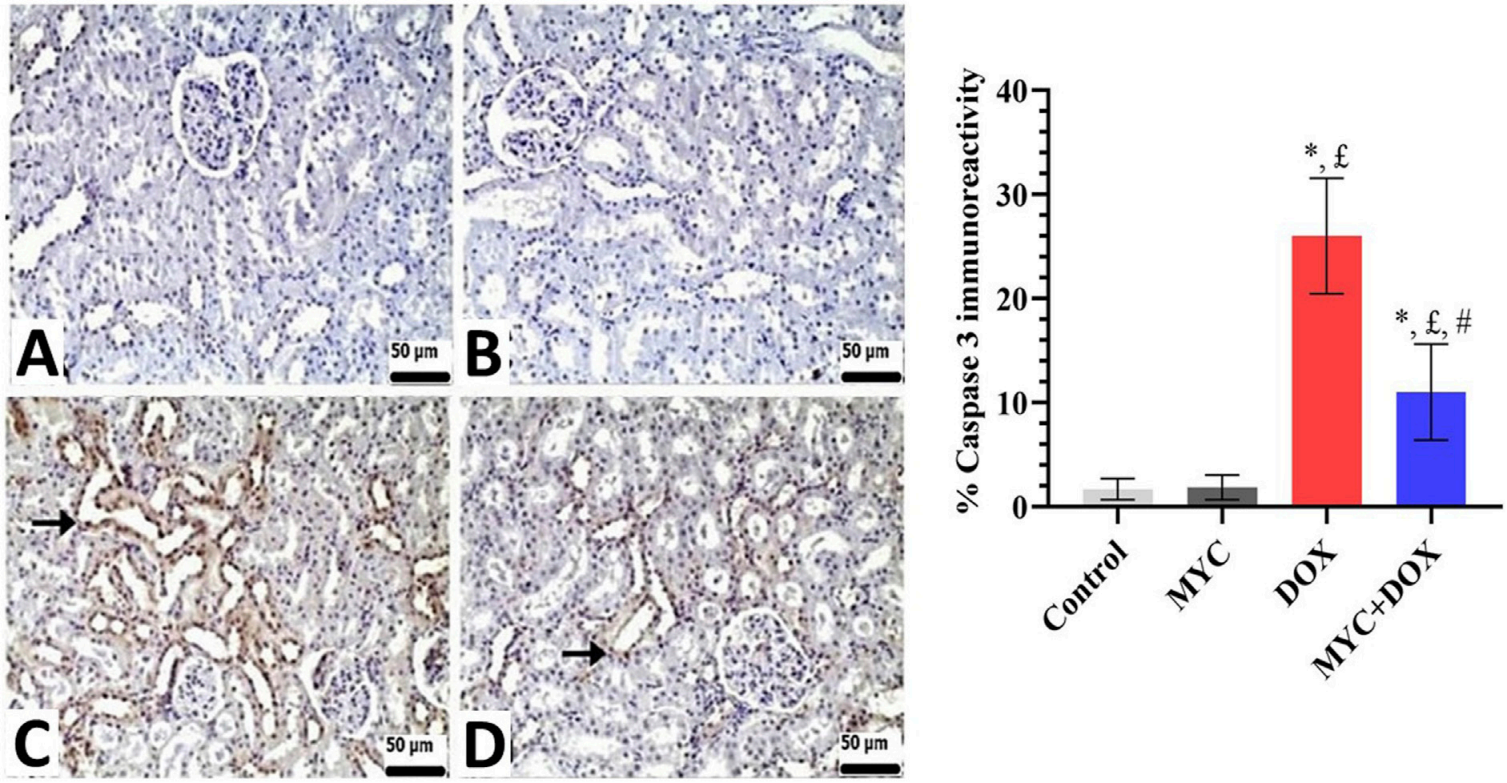
**(A–D)** Immunohistochemical staining of caspase-3 in renal tissue of rats from the control, MYC, DOX, and MYC + DOX. (**A,B)** Control **(A)** and MYC-only **(B)** treated groups demonstrated immunoreactivity for caspase-3. **(C)** DOX group. DOX group showed severe immunopositivity levels of caspase 3. (arrow) **(D)**. MYC + DOX group. Pre-treatment with MYC reverted with these alterations with mild expression levels of caspase-3. Scale bar = 50 µm. Results portrayed mean ± SD of six animals per group. *; p < 0,05 vs control, £; p < 0.05 vs. MYC group, #; p < 0.05 vs. DOX group. DOX, doxorubicin; MYC, myricetin.

**FIGURE 8:**
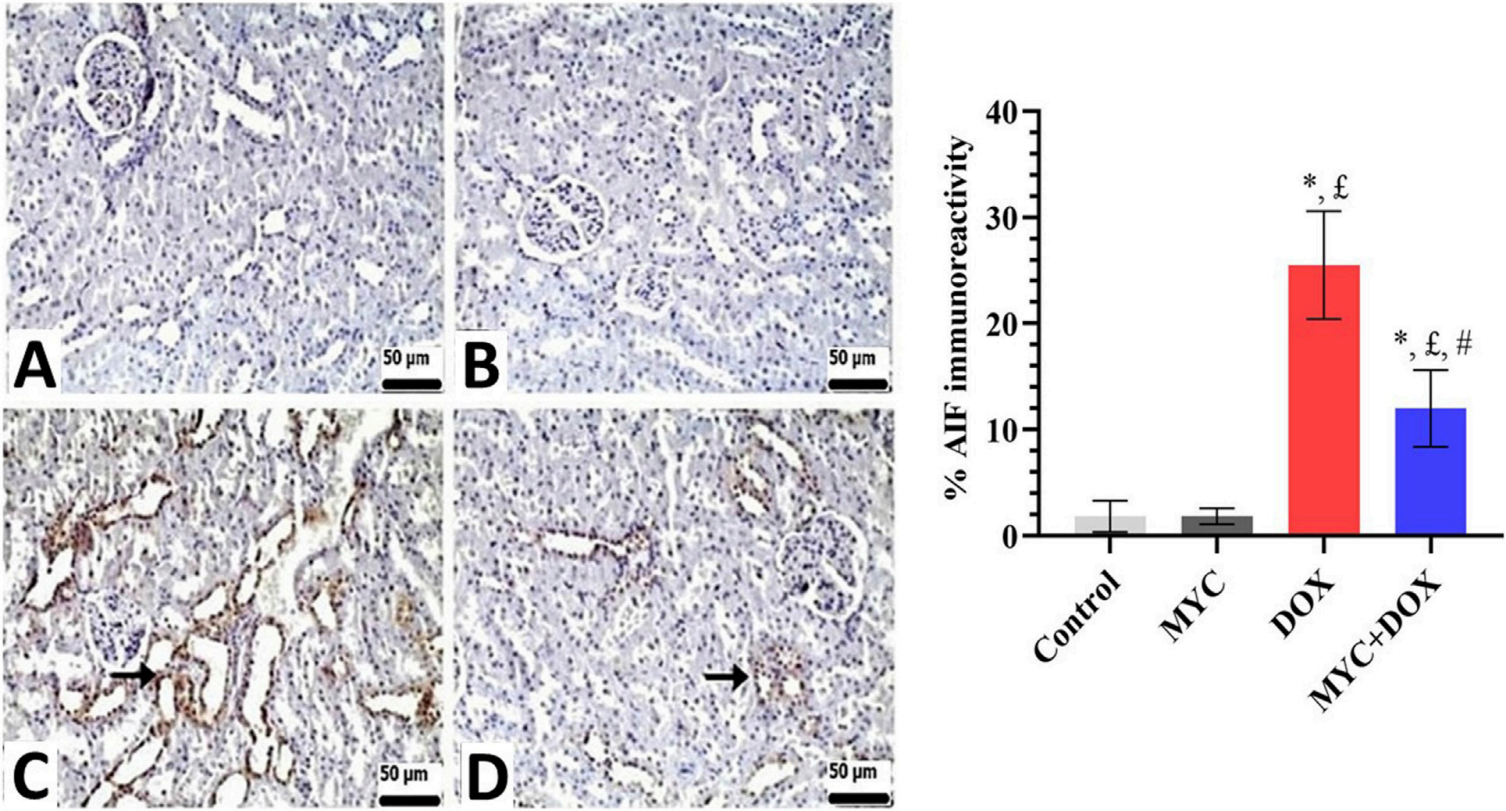
**(A–D)** Immunohistochemical staining of AIF in renal tissue of rats from the control, MYC, DOX, and MYC + DOX. (**A,B)** Control **(A)** and MYC-only **(B)** treated groups demonstrated immunoreactivity for AIF. **(C)** DOX group. DOX group showed severe immunopositivity levels of AIF (arrow) **(D)**. MYC + DOX group. Pre-treatment with MYC reverted with these alterations with mild expression levels of AIF (arrow). Scale bar = 50 µm. Results portrayed mean ± SD of six animals per group. *; p < 0,05 vs control, £; p < 0.05 vs. MYC group, #; p < 0.05 vs. DOX group. AIF, apoptosis-inducing factor; DOX, doxorubicin; MYC, myricetin.

Both the control and MYC groups’ kidney sections showed no discernible caspase-3 or AIF immunopositivity (Figures 7A,B, 8A,B). On the other hand, DOX administration resulted in a high expression of caspase-3 or AIF in comparison with the control group (Figures 7C, 8C). In the DOX group, 26% caspase-3 immunopositivity and 25% AIF immunopositivity were observed. Interestingly, MYC pre-treatment provided diminished immunoreactivity scores of these apoptotic markers in comparison with the DOX-only treated group (p < 0.05; Figures 7D, 8D). caspase-3 immunopositivity decreased by 58% in the MYC + DOX group compared to the DOX group, falling to 11%; AIF immunopositivity decreased by 52%, falling to 12%.

## Discussion

This report aimed to provide scientific proof of the potential use of natural compounds, like MYC, as an effective defense against nephrotoxicity associated with chemotherapy drugs, such as DOX. Nephrotoxicity, a chief aftereffect of DOX treatment, is principally related to oxidative damage and apoptosis in the renal tissues (Xiang et al., 2021). In this report, DOX-induced nephrotoxicity was reported by noticeably elevated renal damage indicators, creatinine, and BUN, morphologically by altering the renal architecture in the form of interstitial nephritis and tubular degeneration. These changes were accompanied by a reduction in antioxidant capacity and elevated signals of renal oxidative stress and proapoptotic markers. Interestingly, MYC pre-treatment effectively restored the renal function biomarkers in serum, maintained the renal histology, and reversed the elevation in the oxidative and apoptotic markers’ levels.

It is well known that serum creatinine and BUN levels are the most generally used indicators reflecting the degree of glomerular damage and renal dysfunction (Gounden et al., 2024). We found that serum BUN and creatinine levels were significantly elevated in the DOX group, results that aligned with previous research. (Cirrik et al., 2023; Owumi et al., 2021). In support of biochemical findings, our histopathological examination revealed severe interstitial nephritis consisting of mononuclear cell infiltration and tubular degeneration in the kidney tissue of rats after DOX administration, which is consistent with the literature data (Owumi et al., 2021; AlAsmari et al., 2022) It is noteworthy that MYC pre-treatment significantly reduced the elevation of creatinine and BUN resulting from DOX administration and improved histological interstitial nephritis and tubular degeneration. These outcomes are similar to those of Yang et al., who reported functional and noticeable structural improvement upon MYC treatment in experimental animals with a urolithiasis model (Yang et al., 2024). The role of MYC in maintaining renal structure and function may be attributed to its antioxidant and free radical scavenging properties.

We further investigated distinctive oxidative and apoptotic markers to determine possible mechanisms underlying the renoprotective actions of MYC in DOX-evoked acute kidney injury. Renal dysfunction in animals exposed to DOX indicates the ability of this drug to decrease antioxidant activity, increase ROS, and activate LPO (Afsar et al., 2020). According to current findings, the occurrence of renal damage in the DOX group was corroborated by changes in renal redox status that displayed a manifest increment in MDA and TOS levels, along with a reduction in GSH, GPx, and TAS profiles, findings that were accompanied by previous studies (Kaymak et al., 2022; Soltani Hekmat et al., 2021). In contrast, MYC pre-treatment inhibited the deleterious effects of DOX-induced oxidative stress in the kidney. Remarkably, MYC significantly reversed the increase in renal MDA and TOS levels while suppressing the decrease GSH, GPx, and TAS levels. Current report findings suggest that the proven potent metal chelation, free radical scavenging, and antioxidant potential of MYC are associated with its ability to reduce DOX-associated oxidative stress observed in renal tissues (Šimunková et al., 2020). Consequently, obtained results from this study not only align with the current body of literature but also highlight the importance of antioxidant pre-treatment regimens as possible protective approaches for organ toxicities caused by DOX.

The heme-containing enzyme MPO plays a role in the oxygen-dependent mechanisms of professional phagocytes’ microbicidal activity (Kisic et al., 2016). MPO activity elevation is thought to be a quantitative indicator of inflammation and a sign of neutrophil infiltration (Rahmani et al., 2018). Moreover, considering that neutrophils play a role in oxidant damage through mechanisms including the MPO system, the formation of MPO-catalyzed oxidative reactions and lipid adducts (with hypochlorous acid as the main oxidant causing tissue damage by phagocytic cells) further magnifies kidney tissue damage (Yagmurca et al., 2004). In the current report, the finding that renal MPO activity was elevated in the DOX-treated rat is meaningful because it readably signed leukocyte accumulation in the kidney tissue. Furthermore, high levels of MPO finding may be an important mechanistic link between oxidation and inflammation in DOX-associated nephrotoxicity, consistent with one study reported by Yagmurca et al. (Yagmurca et al., 2004). Conversely, pre-treatment with MYC, which is characterized by remarkable anti-inflammatory properties (Rahmani et al., 2023), resulted in suppression of DOX-elevated MPO activity. Investigation across a variety of experimental models has constantly manifested MYC’s ability to alleviate the levels of MPO, as also evidenced by the findings of the present research (Shu et al., 2024; Zhao et al., 2013). Together, these results point to a potential protective and therapeutic role for MYC in managing and preventing DOX-induced nephrotoxicity due to its strong anti-inflammatory properties. MYC may be able to reduce the negative effects of DOX on the kidney and maintain renal function while undergoing chemotherapy by regulating the inflammatory response.

DOX administration significantly stimulates the excessive production of ROS, which leads to mitochondrial dysfunction, a reduction in mitochondrial membrane potential, and ultimately, renal apoptosis (Amarasiri et al., 2023; Arunachalam et al., 2022; Moreira et al., 2014; Zhou et al., 2024). The current study revealed that DOX intoxication produced an elevation in Bax levels and caspase-3 expression as well as a reduction in Bcl-2 levels. Interestingly, our experimental interventions demonstrate that MYC pre-treatment disrupts DOX-induced apoptogenic signaling activation. This pre-treatment resulted in a significant improvement, as evidenced by a decrease in Bax and caspase-3 levels and an increase in Bcl-2 levels. These findings suggest the potential for protection against DOX-induced apoptosis. The observed anti-apoptotic effect may be attributable to the well-documented anti-apoptotic properties of MYC (Hassan et al., 2017; Zhang et al., 2018).

In the current report, the level of AIF was also evaluated. AIF is defined as a pro-apoptotic protein that is known to be involved in caspase-independent cell death in mitochondrial apoptosis pathways (Hussar, 2022). Despite the fundamental role of oxidative stress in AIF release from mitochondria, no study has reported the potential role of AIF in DOX-induced renal cell death. However, Moreira et al. demonstrated that DOX-induced the release of AIF from mitochondria in H9c2 cardiomyoblasts, thus implicating AIF in DOX-induced cell death (Moreira et al., 2014). Moreover, the collective data indicate that DOX-induced mitochondrial apoptosis is initiated by an increase in oxidative mediators, which in turn activate pro-apoptotic signals. Noticeably, our findings support the hypothesis that MYC pre-treatment may have a protective effect against DOX-induced nephrotoxicity by suppressing the DOX-induced increase in AIF levels in kidney tissue. There is no report in the literature that MYC has a protective role against AIF-induced apoptosis.

## Conclusion

In summary, the exposure of rats to DOX resulted in remarkable renal toxicity. This finding was substantiated by the detection of impairments in renal histopathology and the results of functional tests. Evidence of this phenomenon was also demonstrated by increased levels of oxidant parameters with decreased levels of endogenous antioxidants. Additionally, elevated oxidative stress activated apoptosis in response to DOX-induced acute kidney damage. A critical finding of this study was the observation that DOX administration resulted in the release of AIF in kidney tissue. MYC, which has demonstrated potent antioxidant activity, led to a reduction in creatinine and BUN levels, resulting in significant improvements in renal function. Furthermore, this pre-treatment effectively rectified the DOX-induced increase in key markers of cellular damage and oxidative stress. MYC was also effective in preventing the release of various apoptogenic factors by hindering excessive oxidant production. The aforementioned properties of MYC suggest that it may be a suitable candidate for the management of DOX-induced nephrotoxicity under experimental conditions. In light of all this information, the combination of DOX with natural products such as MYC may result in reduced DOX concentrations in healthy cells, elevated DOX levels in cancerous cells, and strengthened anti-tumour properties of DOX. Although the current report demonstrated the nephroprotective properties of MYC against DOX-related kidney damage, further confirmatory research is essential to substantiate this claim. Extensive research, particularly including pharmacokinetics, bioavailability, and clinical trials, is needed to bring MYC closer to practical application.

Limitations: The limitations of this study must be taken into consideration when interpreting the results. First off, because the study was based on a preclinical animal model, it might not accurately capture the complexity and diversity of human DOX-induced nephrotoxicity. Secondly, it would be beneficial to illustrate the relationship between the regulation of ROS levels and apoptosis, as well as the bidirectional effect of the agent on tumor cells and normal cells. Therefore, it can aid in the identification of drugs that counteract the adverse effects of chemotherapy and enhance the efficacy of anti-tumor agents. Thirdly, further studies in clinical settings are required to validate the findings of this study. Finally, it is important to note that the single dose of MYC utilized in the present study may not be representative of the optimal protective dose for administration to human subjects. Therefore, further research is necessary to ascertain the optimal dosing regimen for humans.

## Data Availability

The original contributions presented in the study are included in the article/supplementary material, further inquiries can be directed to the corresponding author.
